# Self-Assembly of Homo- and Hetero-Chiral Cyclodipeptides into Supramolecular Polymers towards Antimicrobial Gels

**DOI:** 10.3390/polym14214554

**Published:** 2022-10-27

**Authors:** Beatrice Rosetti, Erica Scarel, Laura Colomina-Alfaro, Simone Adorinni, Giovanni Pierri, Ottavia Bellotto, Kevin Mamprin, Maurizio Polentarutti, Antonella Bandiera, Consiglia Tedesco, Silvia Marchesan

**Affiliations:** 1 Chemical and Pharmaceutical Sciences Department, University of Trieste, 34127 Trieste, Italy; 2 Life Sciences Department, University of Trieste, 34127 Trieste, Italy; 3 Department of Chemistry and Biology “A. Zambelli”, University of Salerno, 84084 Fisciano, Italy; 4 Elettra-Sincrotrone Trieste, S.S. 114 km 163.5, Basovizza, 34149 Trieste, Italy

**Keywords:** cyclo(Leu-Phe), cyclo(Phe-Phe), diketopiperazines, cyclodipeptides, chirality, D-amino acids, gels, antimicrobial, self-assembly, peptides

## Abstract

There is an increasing interest towards the development of new antimicrobial coatings, especially in light of the emergence of antimicrobial resistance (AMR) towards common antibiotics. Cyclodipeptides (CDPs) or diketopiperazines (DKPs) are attractive candidates for their ability to self-assemble into supramolecular polymers and yield gel coatings that do not persist in the environment. In this work, we compare the antimicrobial cyclo(Leu-Phe) with its heterochiral analogs cyclo(D-Leu-L-Phe) and cyclo(L-Leu-D-Phe), as well as cyclo(L-Phe-D-Phe), for their ability to gel. The compounds were synthesized, purified by HPLC, and characterized by ^1^H-NMR, ^13^C-NMR, and ESI-MS. Single-crystal X-ray diffraction (XRD) revealed details of the intermolecular interactions within the supramolecular polymers. The DKPs were then tested for their cytocompatibility on fibroblast cells and for their antimicrobial activity on *S. aureus*. Overall, DKPs displayed good cytocompatibility and very mild antimicrobial activity, which requires improvement towards applications.

## 1. Introduction

Supramolecular polymers have attracted researchers’ attention in recent years due to the simple and low-cost preparation of their building blocks, which are typically small molecules with the capability to self-organize through the establishment of non-covalent interactions [1]. Amino acids and peptides are popular candidates for various applications, such as the mimicry of natural tissues and the extracellular matrix [2], the development of new contrast agents for bioimaging [3], of antimicrobial hydrogels [4,5], and of carriers for the delivery of drugs [6,7,8,9]. Peptides are versatile, and can be used on their own or combined with other components, such as polymers [10,11,12], proteins [13,14], nanoparticles [15] and carbon nanomaterials [16], cages [17], and so on. However, their physicochemical properties need to be fine-tuned for correct self-assembly into supramolecular polymers, and popular derivatizations to confer strategic structural rigidity include the use of aromatic N-caps [18], dehydropeptides [19], and cyclization [20]. In particular, cyclodipeptides are diketopiperazines (DKPs) that are gaining widespread use for their simple preparation, and their self-assembly has been recently reviewed [21,22].

The simplest DKPs are obtained from the cyclization of linear dipeptides [23,24]. Their ability to form supramolecular polymers that yield gels has been the focus of several studies (Table 1). Hydrogels were obtained from cyclodipeptides containing Phe and a hydrophilic amino acid [25]. Aliphatic amino acids proved to be useful building blocks to obtain organogels [26]. The use of aromatic amino acids, such as Trp and Tyr, afforded both hydrogels and organogels [27,28,29], as did cyclo(Phe-Phe) and cyclo(Leu-Phe) [26,30,31,32,33]. Furthermore, the latter was also reported for hydrogelation in physiological solutions, including phosphate-buffered saline (PBS) and cell-culture medium [30,32], thus opening the way to biological uses. This dipeptide showed the ability to fibrillate into a supramolecular polymer based on H-bonding and aromatic interactions that involved β-type conformations [34,35].

These latter two DKPs are also attractive to yield hydrogels with antimicrobial properties, which could potentially find use in the treatment of skin wounds or topical infections. In particular, cyclo(Phe-Phe) demonstrated anti-helminthic [39] and anti-malarial activity [40]. Cyclo(Leu-Phe) is active against important Gram-positive bacteria, fungi, and yeasts: *Bacillus subtilis*, *Staphylococcus aureus*, *Streptomyces viridochromogenes*, *Mucor miehei*, and *Candida albicans* [41]. Cyclo(Leu-Phe) is oxidized into the antimicrobial dehydro-derivative albonoursin by *Streptomyces* cell-free extracts [42], and the biosynthetic pathway has been elucidated [43]. Besides classical antimicrobial mechanisms, DKPs were found to affect bacterial quorum-sensing systems and cell-cell signaling, thus offering an alternative approach against biofilms, thanks to their interference with microbial communication [44]. Finally, homo- and hetero-chiral cyclodipeptides with aromatic sidechains were reported for their anti-biofilm activities against oral pathogens [45].

However, heterochiral cyclodipeptides are seldom studied for their self-assembling ability into gels [27], with none reported thus far as successful examples of gelators. In this study, we thus selected heterochiral cyclo(Leu-Phe) enantiomers and heterochiral cyclo(Phe-Phe) to fill the knowledge gap, and tested their ability to gel, as well as their cytocompatibility in vitro, and their antimicrobial activity on *S. aureus* as compared against the bioactive homochiral cyclo(Leu-Phe) [41].

## 2. Materials and Methods

### 2.1. Materials

Linear dipeptides L-Leu-L-Phe, D-Leu-L-Phe, L-Leu-D-Phe, and D-Phe-L-Phe were synthesized by solid phase and purified by reversed-phase HPLC, following published procedures [46,47]. All inorganic salts were purchased from Carlo Erba (Milan, Italy). *Staphylococcus aureus* was obtained from ATCC (25923). Mueller–Hinton broth was bought from Millipore (Milan, Italy). All the other solvents and reagents were acquired from Merck (Milan, Italy) and they were used as received, without further purification. High-purity Milli-Q water (MQ water) was employed to prepare all solutions and buffers, as it was produced from a Milli-Q Academic System (Millipore RiOs/Origin purification system; St. Louis, MS, USA) with a minimum resistivity of 18.2 MΩcm. Mouse embryonic fibroblasts (NIH-3T3) were kindly provided by the Department of Life Sciences at the University of Trieste, and they were grown in complete Dulbecco’s Modified Eagle Medium (DMEM) supplied with 10% fetal bovine serum (100 U/mL penicillin, 100 mg/mL streptomycin (GIBCO^®^) and 2% antimycotic and antibiotic (GIBCO^®^). The 3-(4,5-dimethylthiazol-2-yl)-2,5-diphenyl-tetrazolium bromide (MTT) was acquired from Merck (Milan, Italy). The microwave (MW)-assisted synthesis was carried out in a Microwave reactor Discover SP–CEM Corporation. The sonicator used was the Branson Ultrasonic 3800 cleaning bath. NMR spectra were acquired on a Varian 400 MHz spectrometer. ESI-MS spectra were acquired on an Agilent 6120 system.

### 2.2. Synthesis of DKPs 1–4

Each linear dipeptide precursor (i.e., L-Leu-L-Phe, D-Leu-L-Phe, L-Leu-D-Phe, or D-Phe-L-Phe) was dispersed inside a MW glass vial at 30 mM with 1.0 mL of Milli-Q water, by ultrasonication in a water bath at 50 °C for a few minutes [23]. The vial was placed in the MW reactor and heated at 180 °C, 250 W, for 30 min. The reaction mixture was then water-filtered to afford DKP1 (73% yield), DKP2 (66%), DKP3 (66%), and DKP4 (60% yield). Spectroscopic characterization data and spectra can be found in the Supplementary Materials file (Figures S1–S17).

### 2.3. Single-Crystal X-ray Diffraction

Single crystals of DKP1 and DKP2 were collected with a loop, cryoprotected by dipping the crystals in glycerol, and stored frozen in liquid nitrogen. The crystals were mounted on the diffractometer at the Synchrotron Elettra (Trieste, Italy), beamline XRD1, using the robot available at the facility. The temperature was kept at 100 K by a stream of nitrogen on the crystals. Diffraction data were collected by rotating the crystal using a synchrotron radiation wavelength of 0.70 Å, rotation interval 0.5°/image, crystal-to-detector distance of 85 mm. Further details can be found in the Supplementary Materials, Figures S18 and S19 and Table S1.

### 2.4. Gelation Tests

In a glass vial, DKPs 1–4 were dissolved in soybean oil to reach a final concentration of 30 mM or above by vortexing, and then heating to 115 °C in an oil bath for 5 min. The DKPs 2–3 gelled immediately at 115 °C. DKP4 led to a dispersion. DKP1 formed a gel only upon cooling to room temperature after several hours (i.e., the sample was prepared in the evening, and the gel was formed the following morning). Photographs were taken at room temperature after 18 h from sample preparation and are shown in Section 3.3. In phosphate-buffered saline (PBS) buffer, DKPs 1–4 were dissolved in DMSO (20% of final volume), and then diluted with 80% PBS. Only DKP1 gelled (minimum gelling concentration, mgc = 5 mM).

### 2.5. Oscillatory Rheology

The soybean gels (0.5 mL) were prepared as described above in Section 2.4 at 30 mM (or 50 mM for DKP1), then the samples were vortexed to a dispersion, then they were transferred onto the rheometer plate (20 mm diameter, flat) and the top plate was lowered (gap = 0.7 mm). A heating/cooling ramp cycle from 25 °C to 115 °C and back (7 °C/min) was then employed. Then, a time sweep analysis was performed for 30 min (1 Hz, 1 Pa), followed by a frequency sweep (1 Pa) and a stress sweep (1 Hz).

### 2.6. MTT Cytotoxicity Assay

NIH-3T3 fibroblasts were seeded (10 k cells/well) on 96-well microplates (Euroclone, tissue-culture grade treated, clear, flat bottom, sterile) in 100 μL of medium (DMEM + 10% fetal serum albumin, 2% antimycotic and antibiotic from GIBCO) and cultured at 37 °C and 5% CO_2_ for 24 h. Next, the medium was removed and exchanged with 100 μL of medium with serial dilutions of each DKP concentration (1.0 μM–0.5 mM) prepared in medium. A total of 1% SDS served as negative control (death). Cells were cultured for 24 h, then 10 μL of the MTT labelling reagent (Sigma, final concentration of 0.5 mL/mL) was added to each well, and the microplate was incubated for 4 h in a humidified chamber (37 °C and 5% CO_2_). Afterwards, 100 μL of the solubilization solution for formazan crystals (lysis buffer, 4 mM HCl + 0.1% IGEPAL in isopropanol) was added to each well, and the microplate was kept at room temperature while shaking (Rocker-shaker MR-12 Biosan, Vetrotecnica, Padova, Italy) for 30 min. The absorbance was read at 570 nm, with a reference wavelength at 690 nm (light scattering), using a multiwell plate reader (TECAN Infinite M1000 Pro). Data are represented as mean ± standard deviation (*n* = 4).

### 2.7. MIC Assay

*S. aureus* was grown overnight in a 3 mL culture in Mueller–Hinton Broth. The day after, a fresh culture was prepared by inoculating 300 µL of O/N culture in 10 mL of 2.1 g/L Mueller–Hinton broth and the culture was grown for 2.0–2.5 h. At this point, the OD_600_ was measured, and the bacterial culture was diluted in 4.2 g/L Mueller–Hinton broth to reach approximately 1 × 10^6^ CFU/mL. DKPs 1–4 were dissolved at 200 μg/mL in sterile water to prepare the serial dilutions in the range 100–12.5 µg/mL that were deposited (50 µL/well) in a sterile 96-well polystyrene microplate U-shaped bottom well (Sarstedt, Numbrecht, Germany). Sterile water was the control sample. Right after, 50 µL of bacterial solution were added per well, to obtain a seeding density of 5 × 10^5^ CFU/mL in a final volume of 100 µL. The plate was incubated overnight at 37 °C and then the absorbance was measured at 600 nm in a plate reader (Synergy H1, BioTek, Santa Clara, CA, USA). Three independent experiments were conducted with at least 3 replicas each. Average values ± standard deviation (*n* = 9) were calculated and plotted in Excel.

## 3. Results

### 3.1. DKP Synthesis and Molecular Characterization

Each DKP (Figure 1) was obtained through a green protocol that involved the cyclization of the linear dipeptide precursor in water [23]. ESI-MS, ^1^H-, and ^13^C-NMR spectra confirmed the product purity and identity (see Supplementary Materials Figures S1–S17).

^1^H-NMR analysis revealed significant upfield shifts (Δ = 0.5–0.6 ppm) for the αCH protons of the heterochiral DKPs, and even greater shifts (Δ = 0.7–1.2 ppm) for the βCH protons of the homochiral DKP1 (Table 2), suggesting the existence of CH-π interactions. This phenomenon has been previously reported for DKPs containing Phe, whereby the aromatic ring was bent on top of the DKP ring [31,48]. This data suggested that, also in this series, DKPs establish intramolecular CH-π interactions with different CH protons depending on the amino acid chirality, i.e., the αCH protons of the heterochiral DKPs 2–4, and the βCH protons of the homochiral DKP1. 

### 3.2. Single-Crystal XRD and Supramolecular Polymers’ Structures

**Figure 2:**
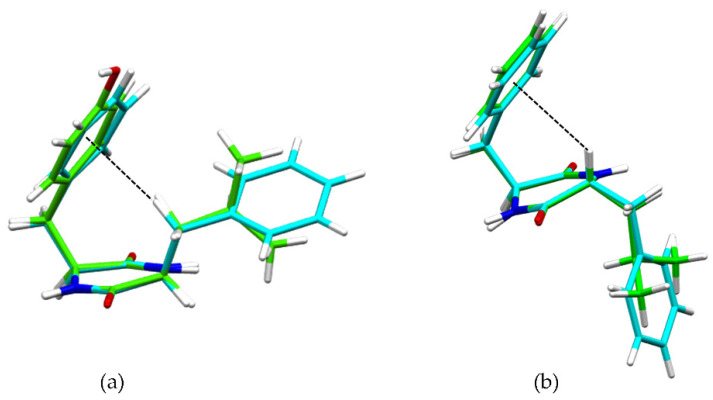
(**a**) Superimposition of single-crystal XRD structures of homochiral cyclo(Tyr-Leu) (green, [49]) and cyclo(Phe-Phe) (cyan, [31]), highlighting the βCH-π interaction (dashed black line). (**b**) Superimposition of single-crystal XRD structures of heterochiral DKP2 (green CCDC 2209459) and DKP4 (cyan, CCDC 2209458) highlighting the αCH-π interaction (dashed black line).

These interactions were confirmed by single-crystal X-ray diffraction (XRD) data for heterochiral DKP2 and DKP4 (Figure 2). Figure 2a shows the βCH-π interaction from the single-crystal XRD data for reference compounds homochiral cyclo(Tyr-Leu) [49] and cyclo (Phe-Phe) [31], while Figure 2b shows the αCH-π interaction for DKP2 (CCDC 2209459) and DKP4 (CCDC 2209458).

**Figure 3:**
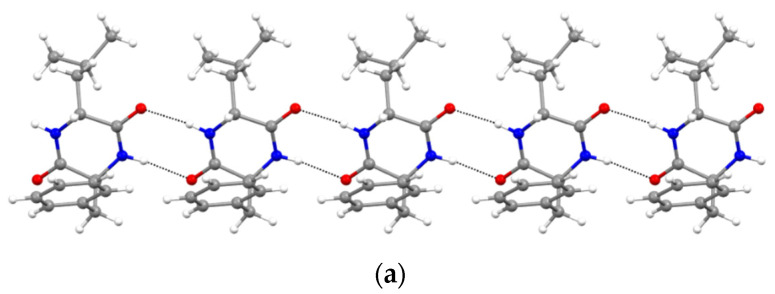

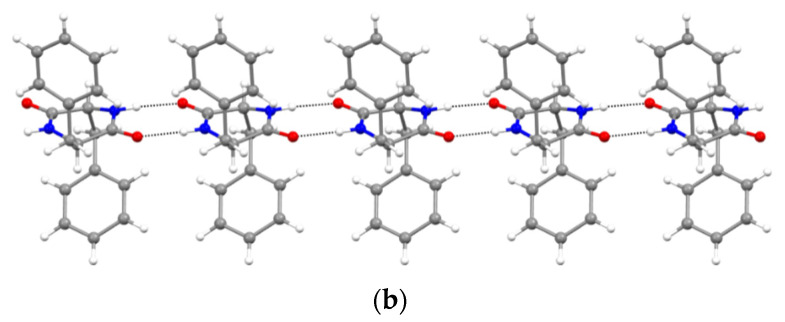
NH∙∙∙OC H-bonded (dashed lines) ribbons in (**a**) DKP2 (CCDC 2209459) and (**b**) DKP4 (CCDC 2209458). Oxygen atoms are shown in red, nitrogen atoms in blue, carbon atoms in grey, and hydrogen atoms in white.

**Figure 4:**
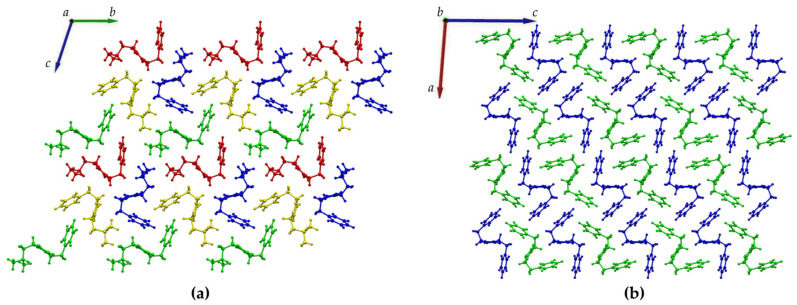
Crystal packing of (**a**) DKP2 (CCDC 2209459) and (**b**) DKP4 (CCDC 2209458) as viewed along the a and b axis, respectively. The independent molecules in the asymmetric unit are depicted in green, blue, red, and yellow in DKP2 and green and blue in DKP4.

Furthermore, XRD analysis revealed the intermolecular interactions that were holding together the supramolecular polymers, and consisted of H-bonds between the amide groups of the stacked DKP rings, and of hydrophobic interactions between adjacent sidechains (Figure 3). In particular, DKP2 crystallizes in a triclinic unit cell (space group P1) with four independent molecules in the asymmetric unit (Figure 4a). The DKP molecules related by symmetry operation interact by means of NH∙∙∙OC H-bonds (Figure 3) defining the typical DKP NH∙∙∙OC H-bonded ribbons. The independent molecules interact mainly through H-bonds and other weak interactions such as CH-π interactions. DKP4 crystallizes in a monoclinic system (space group P2_1_/c) with two chemically equivalent, but crystallographically independent, molecules in the asymmetric unit (Figure 4b). Additionally, in this case, the crystal packing features the typical solid-state arrangement of DKPs characterized by NH∙∙∙OC H-bonded ribbons. The two independent molecules mainly interact through a H-bond involving the carbonyl oxygen atom O2B (C1AH1AA∙∙∙O2B = 2.51 Å, C1AH∙∙∙O2B = 3.254(1) Å, C1AH1AA∙∙∙O2B = 130.8°), CH-π, and π-π interactions. In summary, both crystal structures feature a supramolecular polymer of DKPs aligned along the shortest unit cell axis sustained by NH∙∙∙OC H-bonds.

### 3.3. DKP Self-Assembly and Gelation

**Figure 5:**
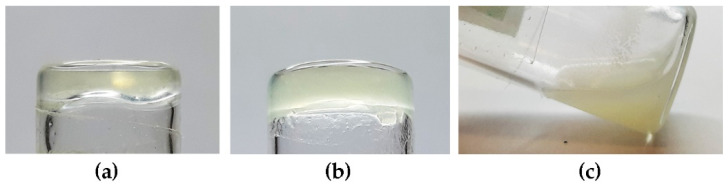
Photographs of inverted-tube tests to probe for the gelation ability of DKPs 1–4 in soybean oil. (**a**) DKP1; (**b**) DKP2 (or DKP3); (**c**) DKP4.

Previous studies revealed that homochiral cyclo(Leu-Phe) or DKP1 and homochiral cyclo(Phe-Phe) formed precipitates in a variety of solvents, spanning from aqueous conditions, to alcohols, and organic solvents [26,33]. Two exceptions were phosphate-buffered saline (PBS) solutions and oil, which allowed to obtain hydrogels [30,31,32] and organogels [26,33], respectively, for both DKPs. We chose DKP1 as reference for self-assembly and gelation in these two solvent systems (Table 3). Heterochiral DKPs 2–4 inevitably led to the formation of precipitates in aqueous conditions, as reported for heterochiral cyclo(Tyr-Tyr) [27]. However, DKPs 2–3 gelled soybean oil with a minimum gelling concentration (mgc) of 30 mM (Figure 5).

**Figure 6:**
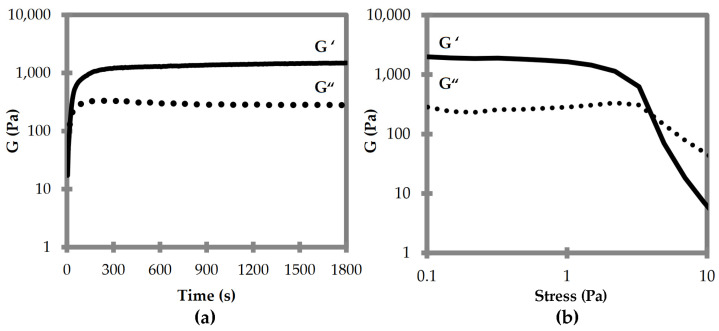
Oscillatory rheological analyses for DKP2 soybean gels. (**a**) Time sweep. (**b**) Frequency sweep. G′ is the elastic or storage modulus. G″ is the viscous or loss modulus.

Oscillatory rheological analyses were thus performed to compare the viscoelastic properties of the two supramolecular polymers composed of DKP1 and DKP2 stereoisomers. Time sweeps for DKPs 2–3 gelation revealed that the viscoelastic moduli G’ and G” reached a plateau after 15 min, with a G’ of 1.5 kPa (Figure 6a). Stress sweeps revealed a gel-to-sol transition occurring at 5 Pa (Figure 6b). Frequency sweeps confirmed the gel nature and its stability, with both G’ and G” being independent from the applied frequency (see Supplementary Materials Figure S20). Unfortunately, all attempts to perform rheological analyses of the homochiral DKP1 gel failed, even at higher concentrations (i.e., 50 mM). It is possible that the material is not very stable, and even minimal stresses due to sample handling lead to the gel-to-sol transition.

Overall, the gelation tests revealed opposite behavior for the DKPs in the aqueous conditions versus oil. This observation could be ascribed to the homochiral DKP being the only one adopting an amphipathic structure with both hydrophobic sidechains pointing on the same side of the DKP ring (Figure 2a), thus exposing the hydrophilic amide groups on the opposite side. This net segregation between hydrophilic and hydrophobic components was found also for the homochiral analog of DKP4 that indeed proved able to form hydrogels [31]. Conversely, heterochiral DKPs 2–3 display the hydrophobic sidechains pointing in opposite directions relative to the DKP ring (Figure 2b), without a net segregation between them and the hydrophilic groups for hydrogelation. In oil, we can expect a substantially different behavior, and indeed while both homochiral and heterochiral DKPs 1–3 gelled, the heterochiral DKPs 2–3 did so more rapidly than DKP1. Therefore, we can conclude that for biological applications that require aqueous environments, DKP1 is the best candidate of this series as it forms hydrogels.

### 3.4. DKP Cytocompatibility

**Figure 7:**
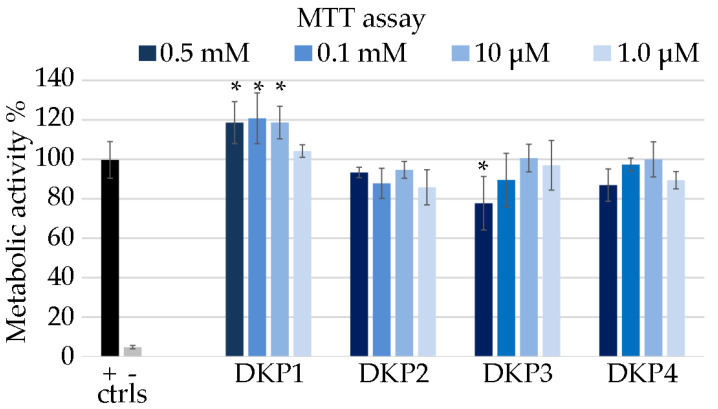
MTT assay on fibroblast cells treated with the DKPs 1–4, or 1% SDS (− ctrl), or untreated (+ ctrl). Metabolic activity % is shown relative to the + ctrl. * denotes *p* < 0.01.

Each DKP was tested for cytocompatibility using the metabolic MTT assay on fibroblast cells [23]. DKPs are natural biomolecules that occur also in foodstuff, with homochiral DKP1 being present in roasted coffee [50] and cocoa nibs [51]. Therefore, they are generally expected to display good cytocompatibility, especially those composed of naturally occurring L-amino acids. Surprisingly, DKP1 significantly increased the metabolic activity of fibroblasts in the concentration range 10–500 μM (Figure 7), and it is possible to ascribe this effect to the enzymatic hydrolysis of the DKP into the corresponding linear dipeptides, which are in fact cell nutrients. Conversely, heterochiral DKPs 2–4 generally yielded absorbance values that were not statistically significantly different relative to the control. DKPs 2–4 demonstrated good cytocompatibility at all tested concentrations, with the worst performance being ascribed to DKP3 at the highest concentration of 0.5 mM, which led to the only statistically significantly lower value (78%) relative to the control. Higher concentrations could not be tested due to insolubility of the DKPs.

### 3.5. DKP Antimicrobial Activity

**Figure 8:**
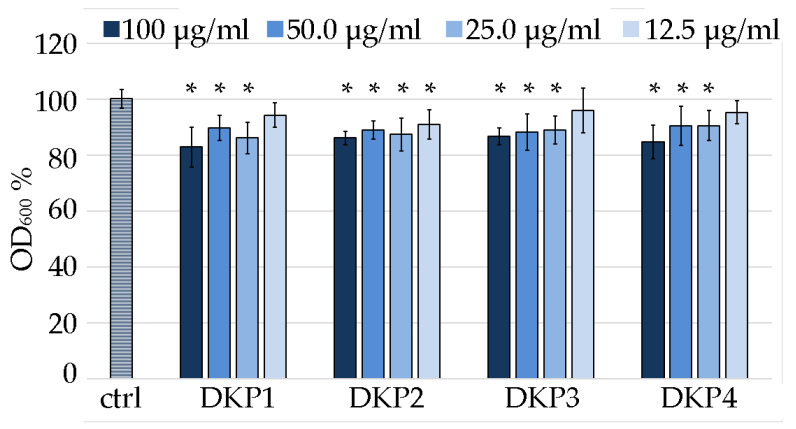
Minimum inhibitory concentration assay on *S. aureus* for DKPs 1–4 measured as OD_600_ normalized to the untreated *S. aureus* (100% control, ctrl). * denotes *p* < 0.01.

DKP1 was reported to exert antimicrobial activity on Gram-positive bacteria, fungi, and yeasts at the concentration of 0.1 mg/mL [41]. In particular, the activity against *S. aureus* is of relevance worldwide, being one of the most frequent pathogenic causes of morbidity and mortality [41]. Although *S. aureus* normally colonizes the human anterior nares, it is an opportunistic pathogen that can also lead to life-threatening bloodstream infections, such as endocarditis and sepsis [52]. Therefore, in this work we chose this bacterium as a model system to identify the minimum inhibitory concentration (MIC) of DKP1 and compare it against the heterochiral DKPs 2–4 (Figure 8).

Considering the DKPs poor solubility, the reportedly effective antimicrobial concentration of 0.1 mg/mL [41] was used as the highest value of reference. Two-fold series dilutions revealed that at 12.5 μg/mL, DKP1 was ineffective, as it led to data that were not statistically significantly different to the control. Analogous results were obtained for DKP3 and DKP4. DKP2 displayed a slightly better performance, as also at 12.5 μg/mL it yielded absorbance values that were statistically significantly different from the control. However, a further two-fold dilution to 6.25 μg/mL was accompanied with a total loss of activity (not shown). Overall, while the reported antimicrobial activity for DKP1 was confirmed, and similar results were obtained for DKPs 2–4, clearly, further derivation studies to obtain more effective inhibition of bacterial growth are needed to enable any practical application. Alternatively, they could be used as vehicles for more potent antimicrobials and tested for any synergistic effects.

## 4. Conclusions

In conclusion, four DKPs were obtained using a green protocol that involves the microwave-assisted cyclization of linear dipeptide precursors in water. All the desired products were characterized by ESI-MS, ^1^H-, and ^13^C-NMR, which confirmed their identity and purity. Single crystals of suitable quality for XRD analysis were obtained for DKP2 and DKP4 and revealed a conformation with the two hydrophobic side chains pointing in opposite directions relative to the DKP ring, as expected for heterochiral cyclodipeptides.

The DKPs’ ability to self-assemble into gelling supramolecular polymers was tested in PBS and soybean oil, as these two solvent systems were reported to yield gels for DKP1, which precipitated in a variety of other conditions. Indeed, one limitation of all the tested DKPs is their limited solubility in both aqueous and organic solvents, thus rendering their handling somewhat challenging. Nevertheless, gels were obtained in soybean oil for DKPs 2–3, thus opening the way to their potential use in topical formulations. However, for biological uses that require aqueous environments, DKP1 is the best candidate of this series as it is the only one that also yielded hydrogels.

The bioactivity of the four DKPs was also tested in fibroblast cells and *S. aureus*. All DKPs displayed overall good cytocompatibility using a metabolic assay on fibroblast cells, as expected for this kind of biomolecules that occur in nature and foodstuffs. The reported antimicrobial activity of DKP1 was confirmed to be very mild on *S. aureus*, and the MIC was determined to correspond to 25 µg/mL. Similar results were obtained for the other DKPs, with only DKP2 displaying a slightly lower MIC of 12.5 µg/mL. Clearly, a significant enhancement of the antimicrobial activity will be needed for any practical application of these compounds as antimicrobials, as well as of their solubility properties, which, thus far, limited the potential use of their supramolecular polymers as gelators. Considering that the dehydro-derivative of DKP1 is also antimicrobial [53], and that dehydro-cyclodipeptides were reported to form supramolecular gelling polymers [19], this class of analogues may offer a potential avenue to further develop these systems towards antimicrobial gels and coatings with better performance.

## Figures and Tables

**Figure 1:**
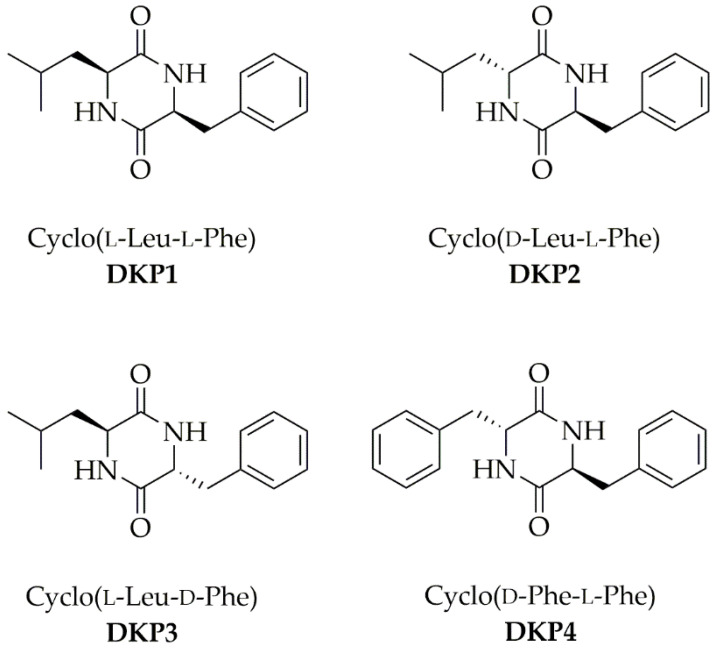
Four cyclodipeptides or DKPs used in this work.

**Table 1:** Gelling DKPs obtained from unprotected dipeptides. Reproduced from [21].

DKP Sequence	Gel Type	Reference
Cyclo(Ala-Gly)	Organogel	[26]
Cyclo(Val-Gly)	Organogel	[26]
Cyclo(Leu-Gly)	Organogel	[26]
Cyclo(Leu-Val)	Organogel	[26]
Cyclo(Leu-Ala)	Organogel	[26]
Cyclo(Leu-Leu)	Organogel	[26]
Cyclo(Phe-Gly)	Organogel	[26]
Cyclo(Phe-Gly)	Hydrogel	[36]
Cyclo(Phe-Val)	Hydrogel	[31]
Cyclo(Phe-Leu)	Hydrogel	[30,32]
Cyclo(Phe-Leu)	Organogel	[26,33]
Cyclo(Phe-Phe)	Hydrogel	[31]
Cyclo(Phe-Phe)	Organogel	[26]
Cyclo(Phe-Cys)	Hydrogel	[25]
Cyclo(Phe-Ser)	Hydrogel	[25]
Cyclo(Phe-Glu)	Hydrogel	[25]
Cyclo(Phe-His)	Hydrogel	[25]
Cyclo(Phe-Lys)	Hydrogel	[25]
Cyclo(Trp-Trp)	Organogel	[28]
Cyclo(Trp-Tyr)	Hydrogel	[29]
Cyclo(Tyr-Tyr)	Hydrogel	[28]
Cyclo(Tyr-Tyr)	Organogel	[28]
Cyclo(Tyr-Lys)	Hydrogel	[37]
Cyclo(Tyr-Lys)	Organogel	[37]
Cyclo(Lys-Glu)	Organogel	[38]

**Table 2:** NMR shifts in deuterated DMSO of αCH and βCH protons of DKPs 1–4. Values in bold indicate upfield shifts due to intramolecular CH-π interactions.

DKP	Sequence	αCH Phe	αCH Leu	βCH_2_ Phe	βCH_2_ Leu
DKP1	Cyclo(L-Leu-L-Phe)	4.16	3.47	3.13, 2.83	**0.76**, **0.12**
DKP2	Cyclo(D-Leu-L-Phe)	4.15	**2.88**	3.13, 2.88	1.45, 1.35
DKP3	Cyclo(L-Leu-D-Phe)	4.15	**2.88**	3.13, 2.88	1.45, 1.35
DKP4	Cyclo(D-Phe-L-Phe)	**3.38**	-	3.00, 2.72	-
-	Cyclo(L-Phe-L-Phe) ^1^	3.95	-	**2.55**, **2.20**	-

^1^ Data from ref. [31].

**Table 3:** Gelation tests for DKPs 1–4. P = precipitate. G = gel.

Solvent	DKP1	DKP2	DKP3	DKP4
PBS	G	P	P	P
Soybean oil	G	G	G	P

## Data Availability

Spectroscopic and crystallographic data is available in the Supplementary Materials file. Further data are available from the authors upon reasonable request.

## Supplementary Materials

The following supporting information can be downloaded at: https://www.mdpi.com/article/10.3390/polym14214554/s1, spectroscopic (Figures S1–S17), crystallographic (Figures S18 and S19 and Table S1), and rheological data (Figure S20). References [54,55,56,57,58,59] are cited in the supplementary materials.
