# Supplementary material for: Self-Assembly of Homo- and Hetero-Chiral Cyclodipeptides into Supramolecular Polymers towards Antimicrobial Gels
Source: Polymers (Basel). 2022 Oct 27;14(21):4554. doi: 10.3390/polym14214554 (PMC9654196; doi:10.3390/polym14214554)
Supplement: Supplementary file 1 [file polymers-14-04554-s001.zip › polymers-1984851-supplementary.pdf]

Supporting Information for:

Self-assembly of homo- and hetero-chiral diketopiperazines into supramolecular polymers towards antimicrobial gels

Beatrice Rosetti [1], Erica Scarel [1], Laura Colomina-Alfaro [2], Simone Adorinni [1], Giovanni Pierri [3], Ottavia Bellotto [2], Kevin Mamprin [1], Maurizio Polentarutti [4], Antonella Bandiera [2], Consiglia Tedesco [3,*] and Silvia Marchesan [1,*]

[1] Chemical and Pharmaceutical Sciences Department, University of Trieste, 34127 Trieste, Italy
[2] Life Sciences Department, University of Trieste, 34127 Trieste, Italy
[3] Chemistry & Biology Department, University of Salerno, 84084 Fisciano (SA), Italy
[4] Elettra Sincrotrone, 34127 Trieste, Italy
* Correspondence: ctedesco@unisa.it, smarchesan@units.it

Table of Contents

1. Cyclo(L-Leu-L-Phe) (**DKP1**) spectroscopic data…………………………………….. 2
2. Cyclo(D-Leu-L-Phe) (**DKP2**) spectroscopic data…………………………………….. 4
3. Cyclo(L-Leu-D-Phe) (**DKP3**) spectroscopic data…………………………………….. 6
4. Cyclo(L-Phe-D-Phe) (**DKP4**) spectroscopic data…………………………………….. 8
5. Single-crystal X-ray diffraction……………………………………………………… 10
6. Oscillatory rheology………………………………………………………………….. 13

1. Cyclo(L-Leu-L-Phe) (**DKP1**) spectroscopic data

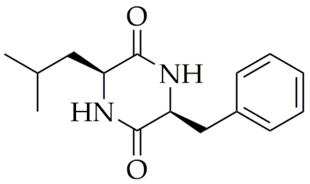

Cyclo(L-Leu-L-Phe)
DKP1

^{1}H NMR (400 MHz, DMSO-*d*$_6$, TMS), δ (ppm): 8.10 (d, *J* = 4.0 Hz, 1H, NH), 8.06 (d, *J* = 4.0 Hz, 1H, NH), 7.30 – 7.12 (m, 5H, ArH), 4.16 (m, 1H, αCH Phe), 3.47 (m, 1H, αCH Leu), 3.13 (dd, *J* = 13.4, 4.0 Hz, 1H, βCH Phe), 2.83 (dd, *J* = 13.4, 5.0 Hz, 1H, βCH Phe), 1.41 (m, 1H, γCH Leu), 0.76 (m, 1H, βCH Leu), 0.63 (d, *J* = 6.5 Hz, 3H, 1 x CH_3), 0.60 (d, *J* = 6.5 Hz, 3H, 1 x CH_3), 0.12 (m, 1H, βCH Leu). **^{13}C NMR** (100 MHz, DMSO-*d*$_6$, TMS), δ (ppm): 167.4, 166.1 (2 x CO); 136.1, 130.4, 128.1, 126.7 (Ar), 55.4, 52.2 (2 x αC); 43.6, 38.4 (2 x βC); 22.9, 22.8, 21.4 (1 x γC, 2 x δC). MS (ESI): m/z 261.1 $(M+H)^+$.

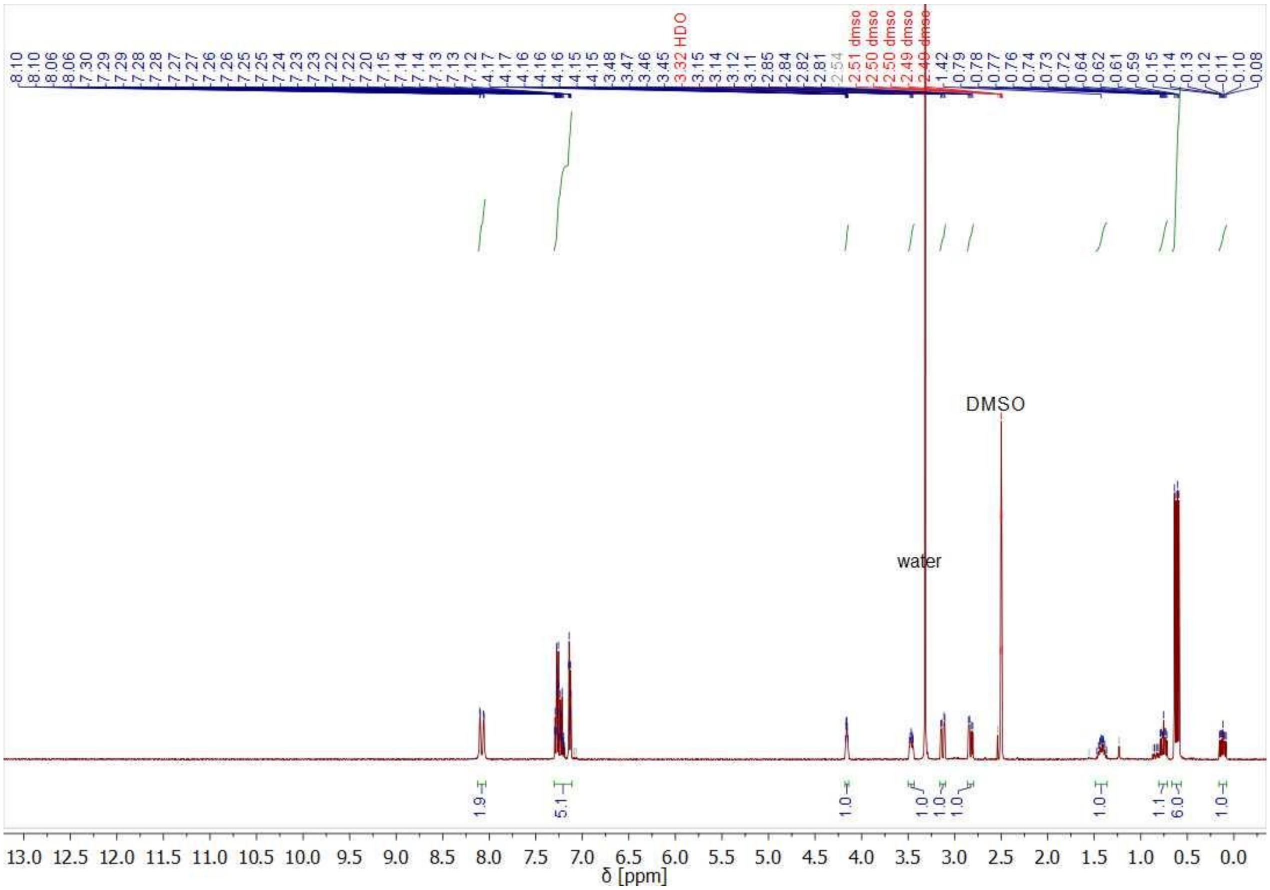

Figure S1. ^{1}H-NMR spectrum of DKP1.

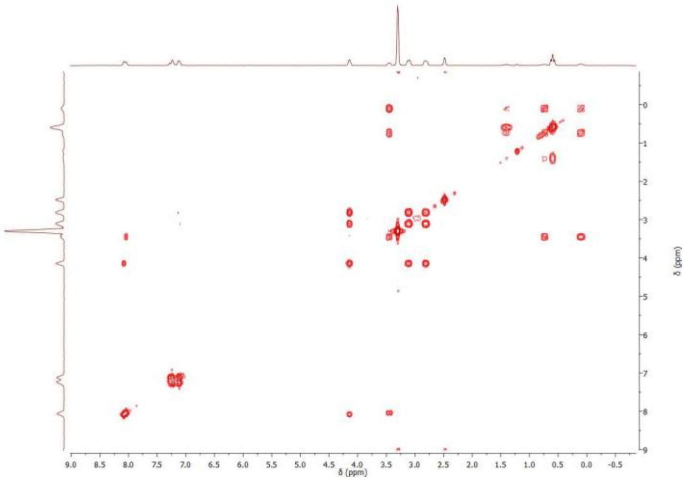

Figure S2. gCOSY 2D-NMR spectrum of DKP1.

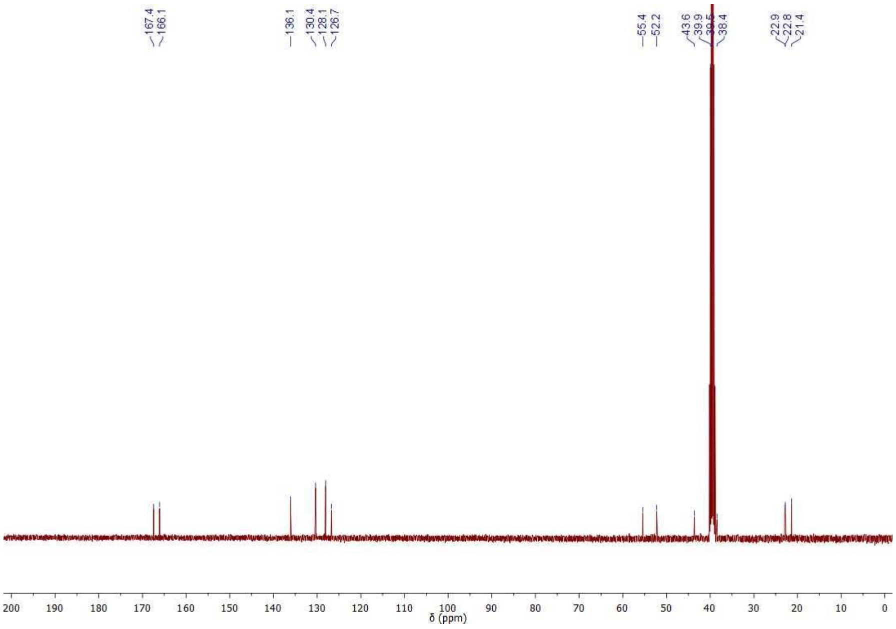

Figure S3. ^{13}C-NMR spectrum of DKP1.

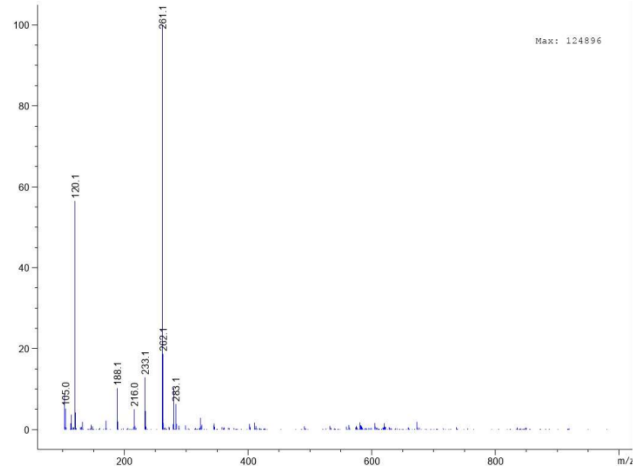

Figure S4. ESI-MS spectrum of DKP1 (positive ion mode).

2. Cyclo(D-Leu-L-Phe) (**DKP2**) spectroscopic data

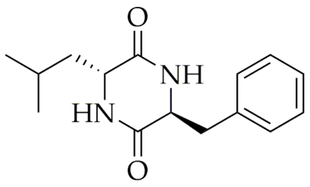

Cyclo(D-Leu-L-Phe)
DKP2

^{1}**H NMR** (400 MHz, CD_3OD, TMS), δ (ppm): 8.06 (d, J = 4.0 Hz, 1H, NH), 8.00 (s(br), 1H, NH), 7.29 – 7.18 (m, 5H, ArH), 4.15 (m, 1H, αCH Phe), 3.13 (dd, J = 13.6, 4.1 Hz, βCH Phe), 2.88 (m, 2H, αCH Leu, βCH Phe), 1.71 (m, 1H, γCH Leu), 1.45 (m, 1H, βCH Leu), 1.35 (m, 1H, βCH Leu), 0.77 (d, 3H, J = 6.6 Hz, 1 x $γCH_3$ Leu), 0.73 (d, 3H, J = 6.6 Hz, 1 x $γCH_3$ Leu). ^{13}**C NMR** (100 MHz, DMSO-d_6, TMS), δ (ppm): 168.3 , 167.4(2 x CO); 136.2, 130.1, 128.0, 126.6 (His); 55.2, 51.8 (2 x αC); 41.4, 38.1 (2 x βC), 23.4 (γC), 22.7, 21.8 (2 x δC). **MS (ESI)**: m/z 261.1 $(M+H)^{+}$.

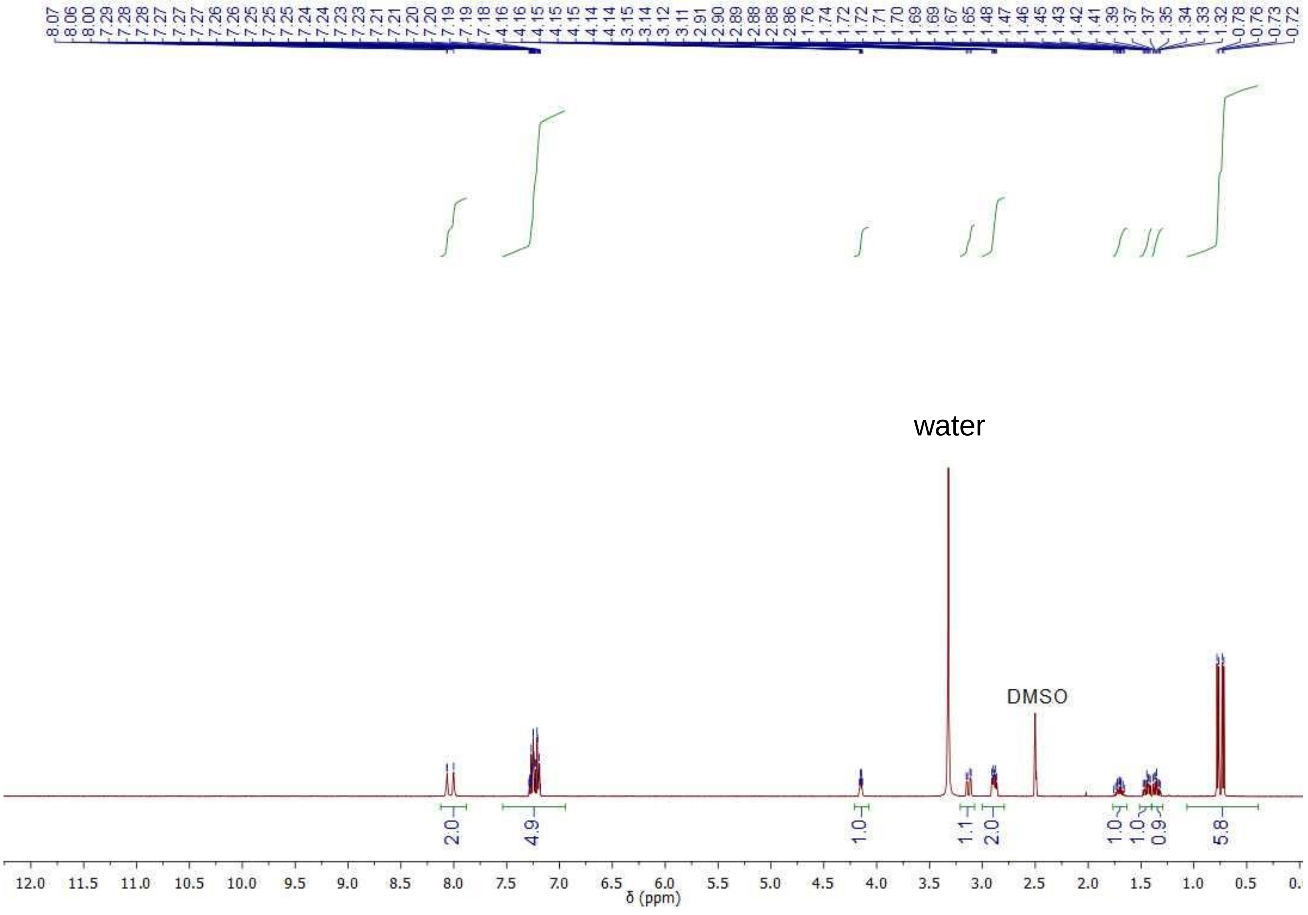

Figure S5. ^{1}H-NMR spectrum of DKP2.

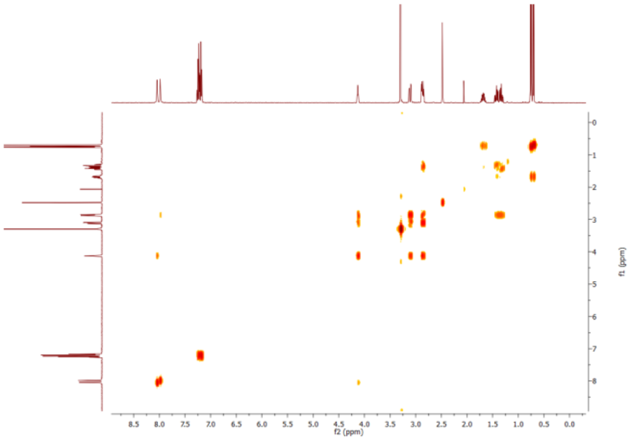

Figure S6. gCOSY 2D-NMR spectrum of DKP2.

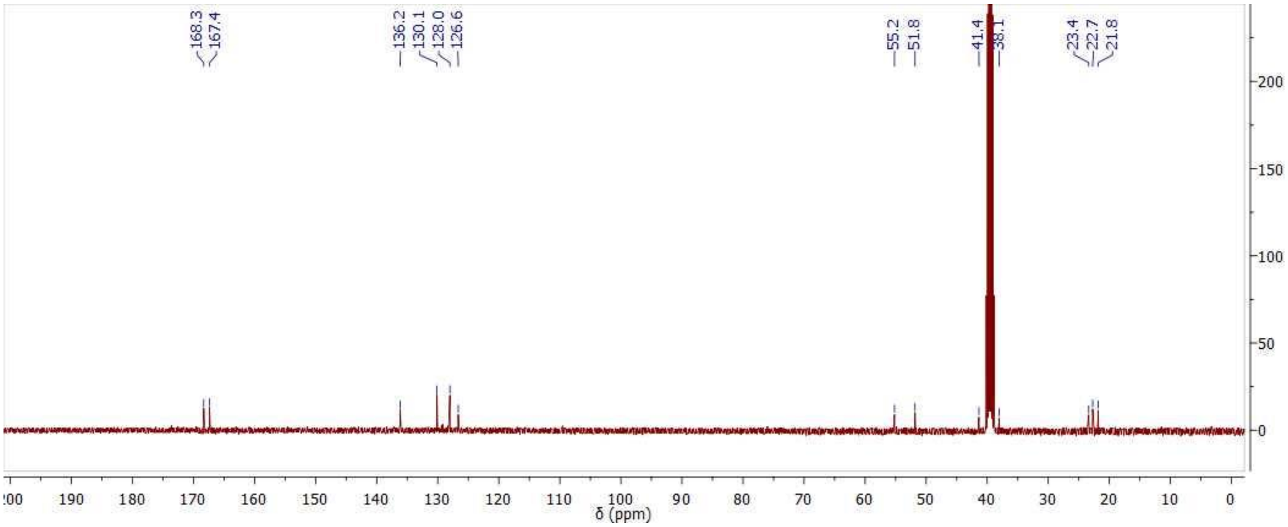

Figure S7. ^{13}C-NMR spectrum of DKP2.

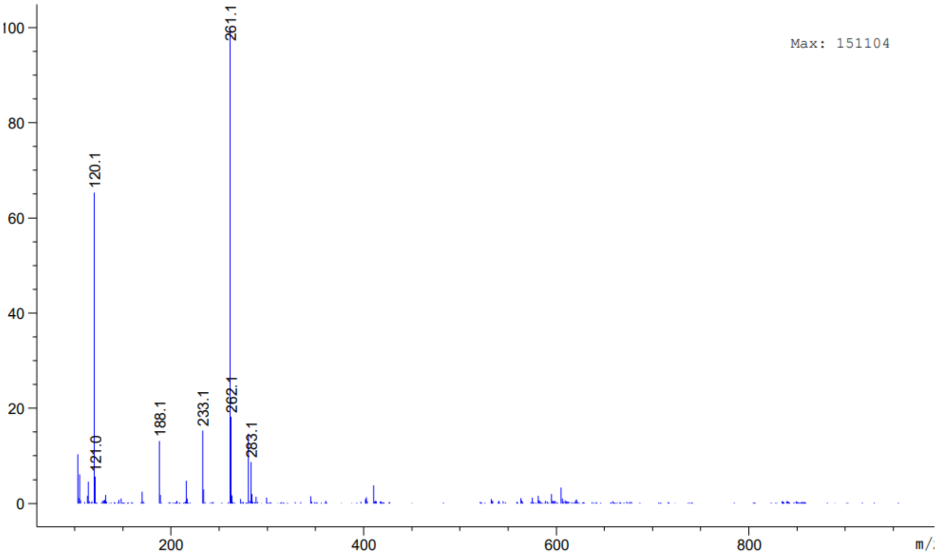

Figure S8. ESI-MS spectrum of DKP2 (positive ion mode).

3. Cyclo(L-Leu-D-Phe) (**DKP3**) spectroscopic data

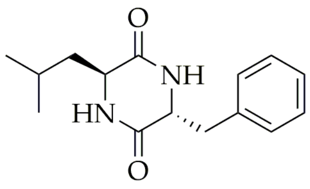

Cyclo(L-Leu-D-Phe)
DKP3

^{1}H NMR (400 MHz, CD_3OD, TMS), δ (ppm): 8.06 (d, *J* = 4.0 Hz, 1H, NH), 8.00 (s(br), 1H, NH), 7.29 – 7.18 (m, 5H, ArH), 4.15 (m, 1H, αCH Phe), 3.13 (dd, *J* = 13.6, 4.1 Hz, βCH Phe), 2.88 (m, 2H, αCH Leu, βCH Phe), 1.71 (m, 1H, γCH Leu), 1.45 (m, 1H, βCH Leu), 1.35 (m, 1H, βCH Leu), 0.77 (d, 3H, *J* = 6.6 Hz, 1 x γCH_3 Leu), 0.73 (d, 3H, *J* = 6.6 Hz, 1 x γCH_3 Leu). **^{13}C NMR** (100 MHz, DMSO-d_6, TMS), δ (ppm): 168.3, 167.4 (2 x CO); 136.2, 130.1, 128.0, 126.6 (Ar),55.2, 51.8 (2 x αC); 41.4, 38.1 (2 x βC); 23.4 (γC); 22.7, 21.8 (2 x δC). MS (ESI): m/z 261.0 $(M+H)^+$.

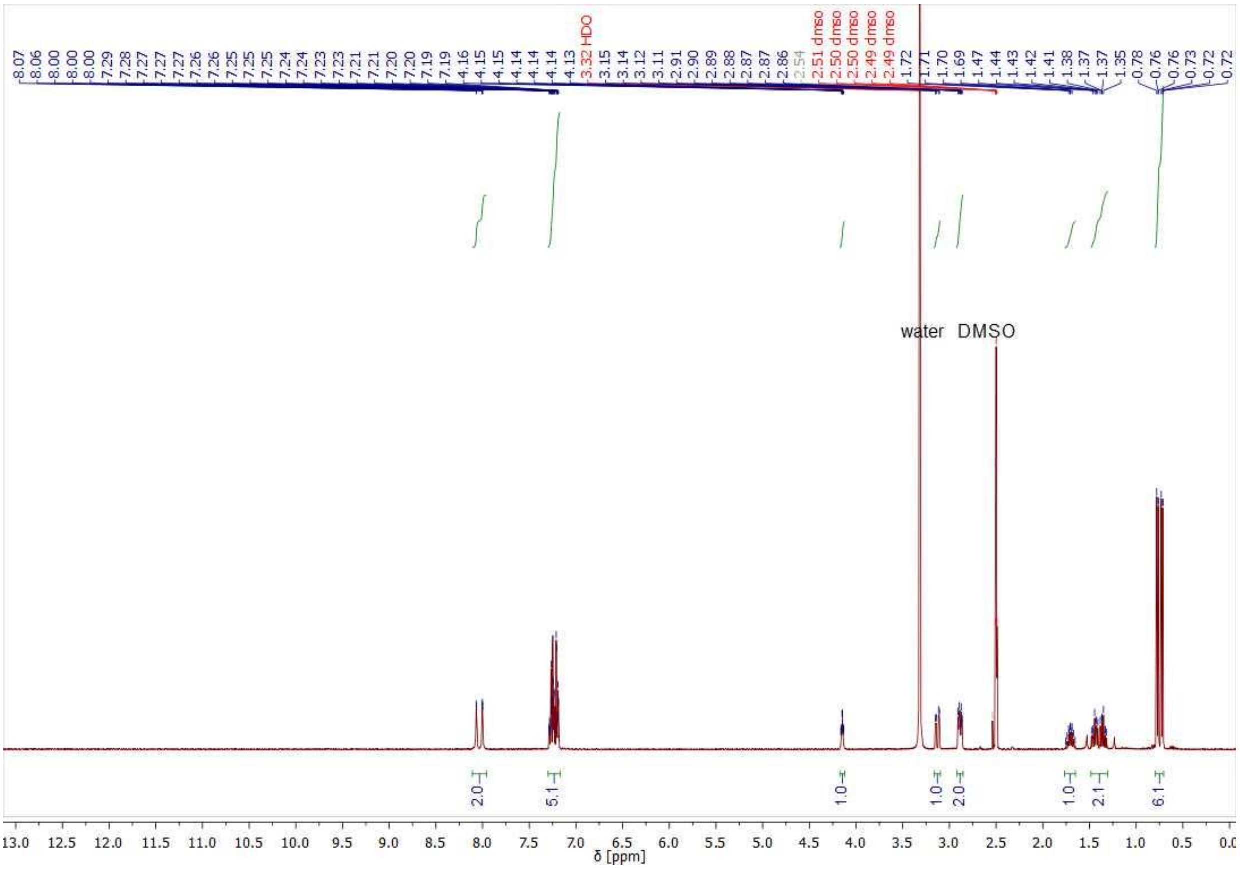

Figure S9. ^{1}H-NMR spectrum of DKP3.

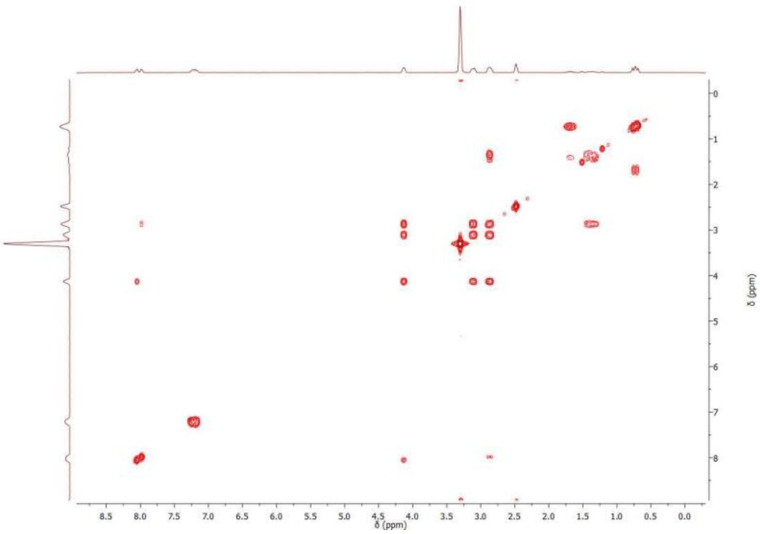

Figure S10. gCOSY 2D-NMR spectrum of DKP3.

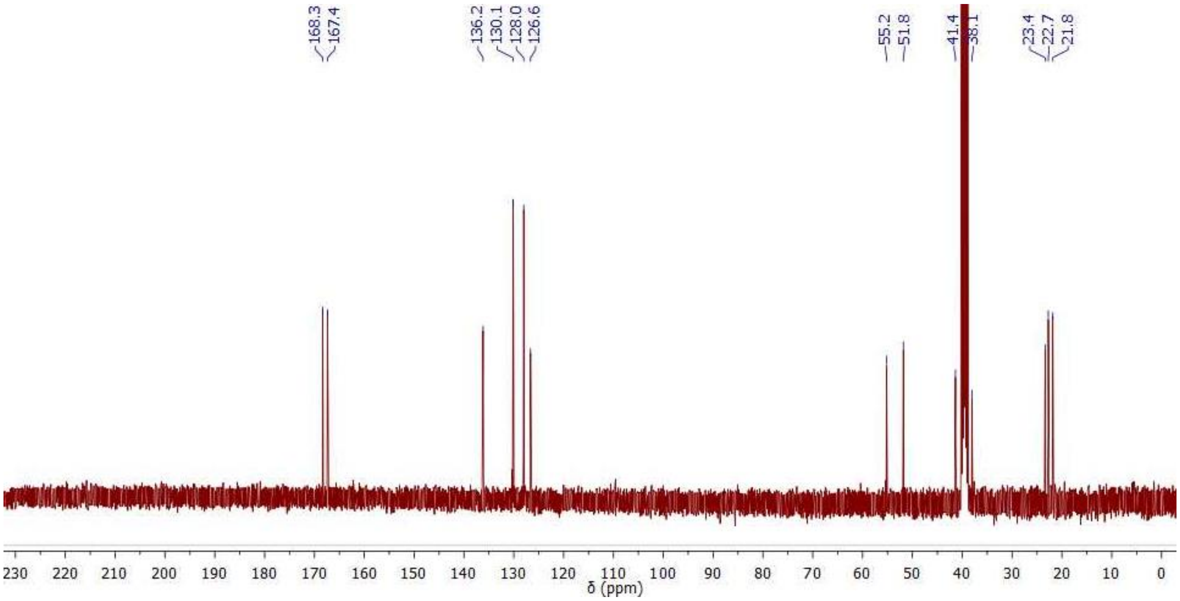

Figure S11. ^{13}C-NMR spectrum of DKP3.

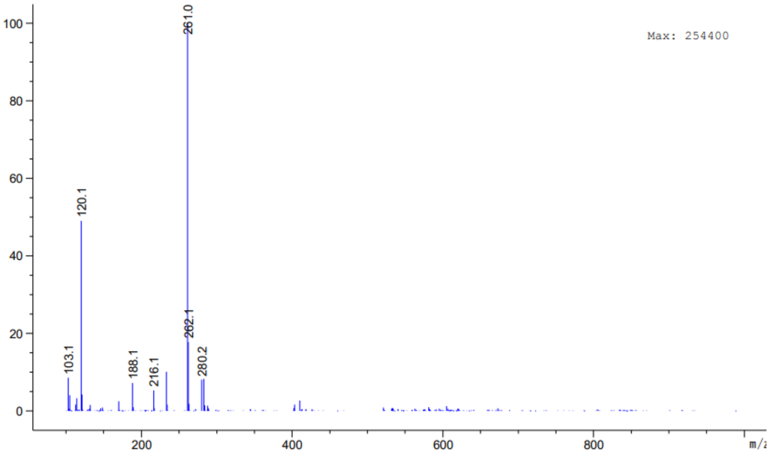

Figure S12. ESI-MS spectrum of DKP3 (positive ion mode).

4. Cyclo(D-Phe-L-Phe) (**DKP4**) spectroscopic data

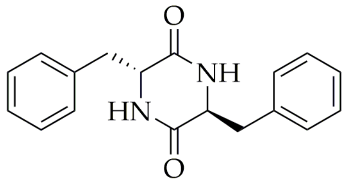

Cyclo(D-Phe-L-Phe)
DKP4

Figure S13. DKP4.

^{1}H NMR (400 MHz, DMSO-d_6, TMS), δ (ppm): 8.04 (s, 2H, NH), 7.27 – 7.10 (m, 10H, ArH), 3.38 (m, 2H, αCH), 3.00 (dd, J = 13.6, 3.6 Hz, 2H, βCH), 2.72 (dd, J = 13.6, 5.2 Hz, 2H, βCH). **^{13}C NMR** (100 MHz, CD_3OD, TMS), δ (ppm): 167.3 (2 x CO); 136.4, 130.5, 128.4, 127.1 (Ar); 55.1 (2 x αC); 38.2 (2 x βC). **MS (ESI):** m/z 295.0 $(M+H)^+$, 317.0 $(M + Na)^+$.

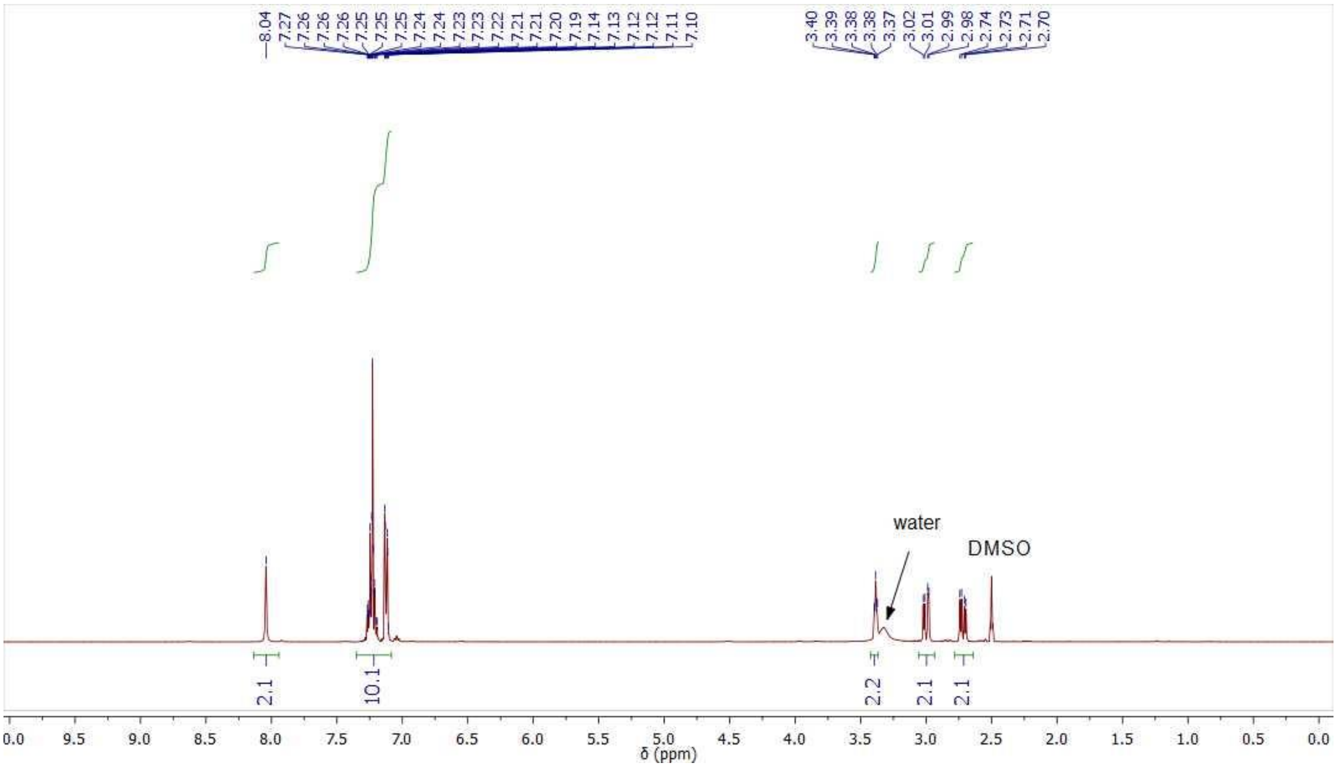

Figure S14. ^{1}H-NMR spectrum of DKP4.

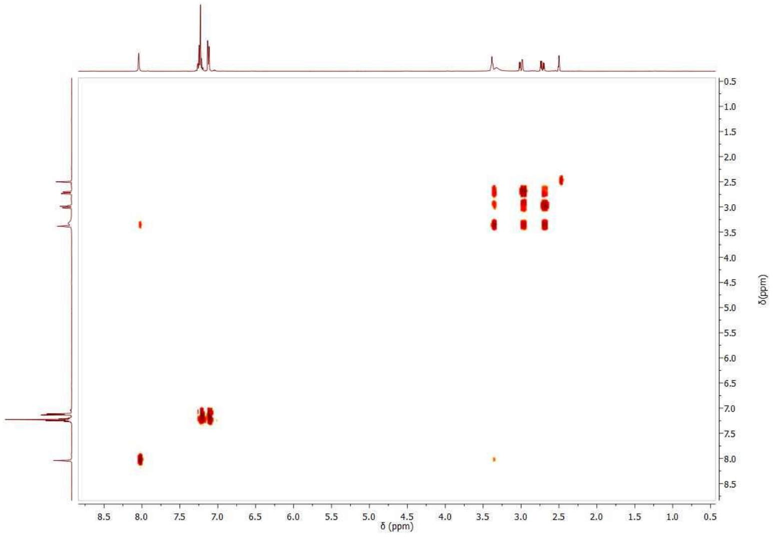

Figure S15. gCOSY 2D-NMR spectrum of DKP4.

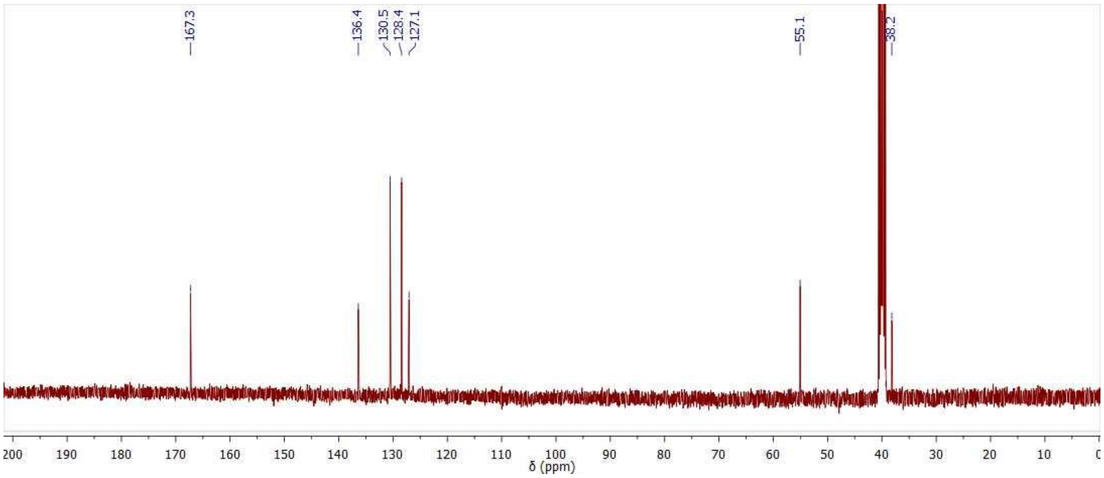

Figure S16. ^{13}C-NMR spectrum of DKP4.

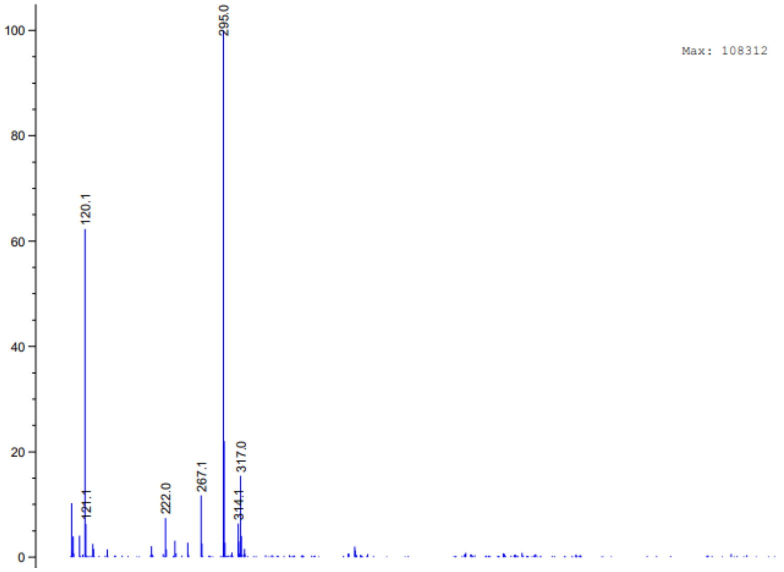

Figure S17. ESI-MS spectrum of DKP4 (positive ion mode).

5. Single-crystal X-ray diffraction

DKP2 (CCDC 2209459) and DKP4 (CCDC 22094598)

Crystals of DKP2 and DKP4 were mounted on the diffractometer at the synchrotron Elettra, Trieste (Italy), beamline XRD1 and measured at 100 K. Data collection were performed using synchrotron radiation (λ = 0.7000 Å) with the rotating crystal method (0.5°/image) for a total of 720 images for **DKP2** and 434 for **DKP4**. Data indexing were performed using MOSFLM,[1] while space groups were determined using POINTLESS.[2] The software AIMLESS[3] was used for scaling the data. The structures were solved using the software SHELXT[4] and refined through full matrix least-squares based on F^2 using the programs SHELXL[5] and OLEX2[6] as a GUI.

Non-hydrogen atoms were refined anisotropically, whereas hydrogen atoms were geometrically positioned and included in structure factor calculations but not refined.

ORTEP diagrams (Figs. S18-19) were drawn using OLEX2. In Table S1 are reported relevant the crystallographic data.

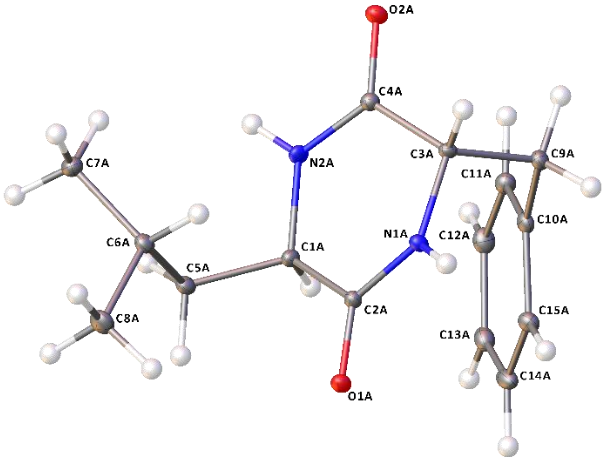

(a)

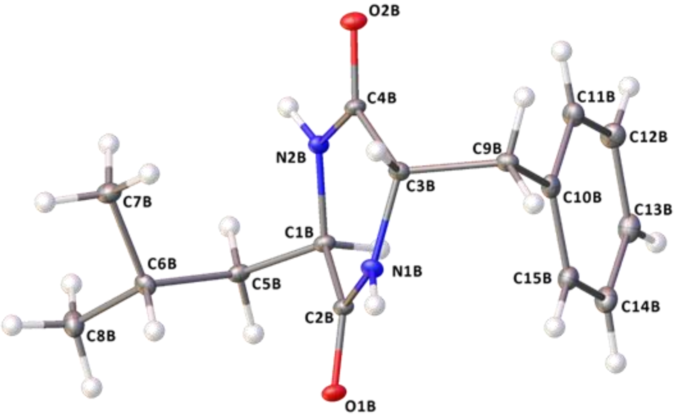

(b)

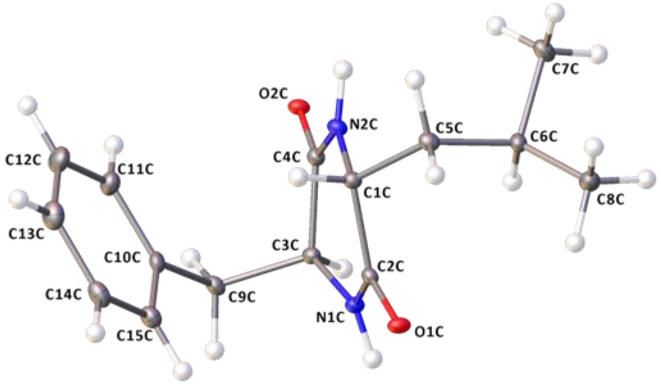

(c)

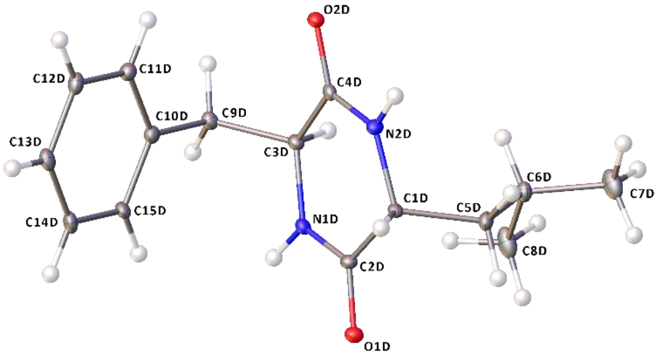

(d)

Figure S18. ORTEP diagrams of DKP2. The four independent molecules in the asymmetric unit are reported. Ellipsoid are drawn at 20% probability level. Atom types: C grey, H white, O red, N blue.

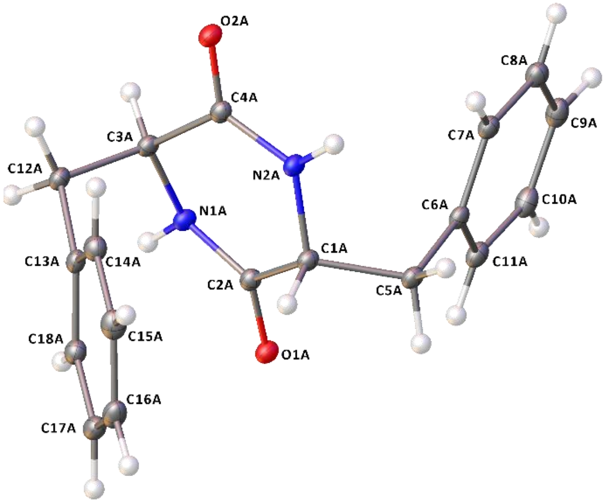

(a)

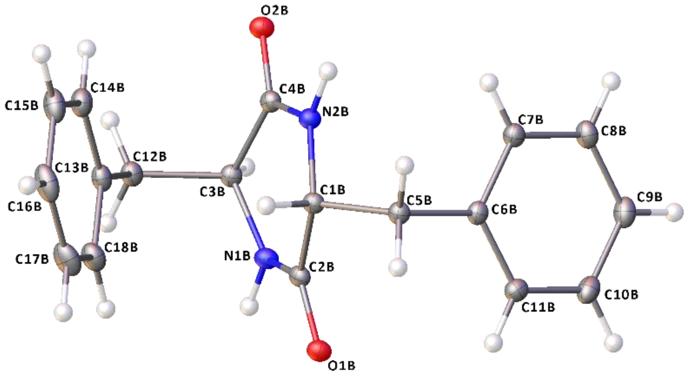

(b)

Figure S19. ORTEP diagrams of DKP4. The two independent molecules in the asymmetric unit are reported. Ellipsoid are drawn at 20% probability level. Atom types: C grey, H white, O red, N blue.

Table S1. Relevant crystallographic data for the crystal structures DKP2 and DKP4.

	**DKP2 (CCDC 2209459)**	**DKP4 (CCDC 2209458)**
**T (K)**	100	100
**Formula**	$C_{15}H_{20}N_2O_2$	$C_{18}H_{18}N_2O_2$
**Formula weight**	260.33	294.34
**System**	triclinic	monoclinic
**Space group**	*P*1	$P2_1/c$
a **(Å)**	6.0860(12)	20.583(4)
b **(Å)**	13.994(3)	6.0840(12)
c **(Å)**	17.283(4)	23.864(5)
α **(°)**	107.61(3)	90
β **(°)**	99.53(3)	94.07(3)

**γ (°)**	91.31(3)	90
**V (Å^3)**	1379.4(5)	2980.9(10)
**Z**	4	8
**D_x (g cm^{-3})**	1.254	1.312
**λ (Å)**	0.70000	0.70000
**μ (mm^{-1})**	0.081	0.084
**F_{000}**	560.0	1248.0
**R1 (I > 2σI)**	0.0816(12209)	0.0729(6502)
**$_wR_2$**	0.2526(14325)	0.2103(8664)
**N. of param.**	694	287
**GooF**	1.055	1.042
**ρ_{min}, ρ_{max} (eÅ^{-3})**	-0.49, 0.52	-0.43, 0.63

References

[1] T. G. G. Battye, L. Kontogiannis, O. Johnson, H. R. Powell and A. G. W. Leslie, *Acta Crystallogr., Sect. D*, 2011, **67**, 271−281.

[2] P. R. Evans, *Acta Crystallogr., Sect. D*, 2006, **62**, 72−82.

[3] P. R. Evans and G. N. Murshudov, *Acta Crystallogr., Sect. D*, 2013, **69**, 1204−1014.

[4] Sheldrick, G. M., *Acta Crystallogr., Sect. A*, 2015, **71**, 3−8.

[5] Sheldrick, G. M., *Acta Crystallogr., Sect. C*, 2015, **71**, 3−8.

[6] O. V. Dolomanov, L. J. Bourhis, R. J. Gildea, J. A. K. Howard and H. Puschmann, *J. Appl. Cryst.*, 2009, **42**, 339−341.

6. Oscillatory rheology

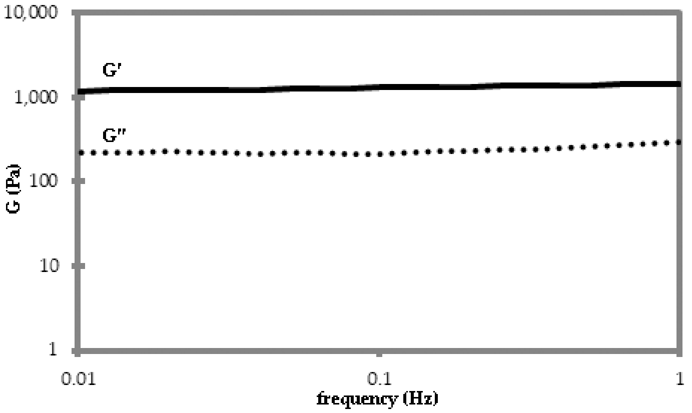

Figure S20. Frequency sweep analysis of DKP2 gel in soybean oil.
